# Cardiac tamponade caused by a ruptured coronary aneurysm treated with open-chest hemostasis after esophageal cancer surgery: a case report

**DOI:** 10.1093/jscr/rjae555

**Published:** 2024-08-28

**Authors:** Makoto Hasegawa, Tomohiro Kikuchi, Hiroki Yago, Dai Mitsui, Akira Matsuishi, Hideaki Tsumuraya, Akinao Kaneta, Hajime Matsuida, Azuma Nirei, Hiroyuki Hanayama, Zenichiro Saze, Shinya Takase, Koji Kono

**Affiliations:** Department of Gastrointestinal Tract Surgery, Fukushima Medical University School of Medicine, Fukushima, Japan; Department of Gastrointestinal Tract Surgery, Fukushima Medical University School of Medicine, Fukushima, Japan; Department of Gastrointestinal Tract Surgery, Fukushima Medical University School of Medicine, Fukushima, Japan; Department of Gastrointestinal Tract Surgery, Fukushima Medical University School of Medicine, Fukushima, Japan; Department of Gastrointestinal Tract Surgery, Fukushima Medical University School of Medicine, Fukushima, Japan; Department of Gastrointestinal Tract Surgery, Fukushima Medical University School of Medicine, Fukushima, Japan; Department of Gastrointestinal Tract Surgery, Fukushima Medical University School of Medicine, Fukushima, Japan; Department of Gastrointestinal Tract Surgery, Fukushima Medical University School of Medicine, Fukushima, Japan; Department of Gastrointestinal Tract Surgery, Fukushima Medical University School of Medicine, Fukushima, Japan; Department of Gastrointestinal Tract Surgery, Fukushima Medical University School of Medicine, Fukushima, Japan; Department of Gastrointestinal Tract Surgery, Fukushima Medical University School of Medicine, Fukushima, Japan; Department of Cardiovascular Surgery, Fukushima Medical University School of Medicine, 1 Hikarigaoka, Fukushima 960-1295, Japan; Department of Gastrointestinal Tract Surgery, Fukushima Medical University School of Medicine, Fukushima, Japan

**Keywords:** cardiac tamponade, esophageal cancer, adult, coronary aneurysm rupture

## Abstract

Cardiac tamponade is a rare postoperative complication of esophagectomy, with no previous reports of association with coronary artery aneurysm rupture. We present a case of cardiac tamponade caused by coronary aneurysm rupture following esophageal cancer surgery. A 68-year-old man with no history of heart disease underwent robotic subtotal esophagectomy for esophageal squamous cell carcinoma. He experienced intermittent chest pain on postoperative day (POD) 17. Echocardiography revealed increasing pericardial fluid, and pericardiocentesis on POD 34 revealed bloody pericardial fluid. Contrast-enhanced computed tomography and coronary angiography revealed a ruptured coronary aneurysm causing cardiac tamponade. Emergency surgery with a median sternotomy achieved hemostasis, and the patient recovered successfully. Cardiac tamponade after esophageal surgery, particularly from coronary aneurysm rupture, is rare. Prompt diagnosis and treatment are crucial for patient survival. Despite its risks, median sternotomy was effective in achieving rapid hemostasis and patient recovery in this case.

## Introduction

Esophageal cancer is not only difficult to cure, but can also cause various complications after surgery. Major complications include pulmonary complications, anastomotic leakage, and anastomotic stenosis [1]. Arrhythmias such as atrial fibrillation are common in 35.7% of postoperative esophageal cancer patients, whereas cardiac tamponade is very rare (0.36%) [2]. We present a case of cardiac tamponade resulting from coronary artery aneurysm rupture after esophageal cancer surgery.

## Case report

A 68-year-old man was incidentally discovered to have an esophageal tumor in the 28–35 cm incisor region on upper gastrointestinal endoscopy. He had no history of heart or collagen disease, or other medical conditions. He had a history of smoking 20 cigarettes per day until 68 years of age and drinking 0.7 units of alcohol per day.

**Figure 1:**
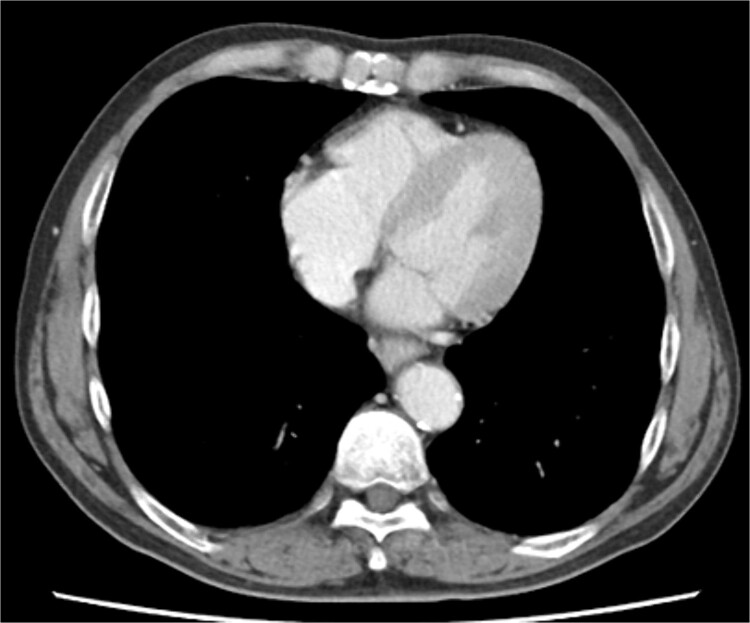
Preoperative contrast-enhanced computed tomography shows no evidence of pericardial effusion, or significant coronary aneurysm.

Biopsy confirmed the diagnosis of squamous cell carcinoma. Contrast-enhanced computed tomography (CT) showed wall thickening in the lower thoracic esophagus. No enlarged lymph nodes and distant metastases were found. There was no evidence of pericardial effusion, or significant coronary aneurysm (Fig. 1). No abnormal findings were observed on 12-lead electrocardiography or echocardiography. Spirometry revealed obstructive ventilatory impairment, and thus, long-acting beta-agonist and muscarinic antagonist inhalation were initiated.

**Figure 2:**
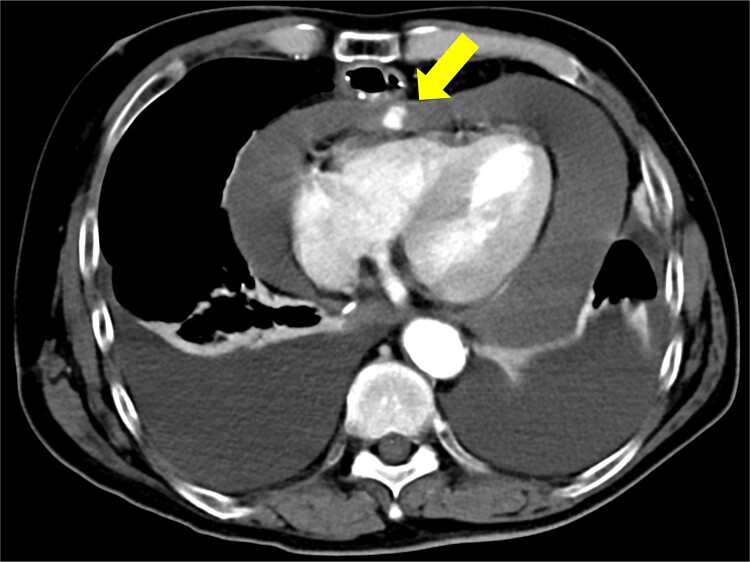
Contrast-enhanced computed tomography shows contrast extravasation in the anterior right ventricle (arrow).

**Figure 3:**
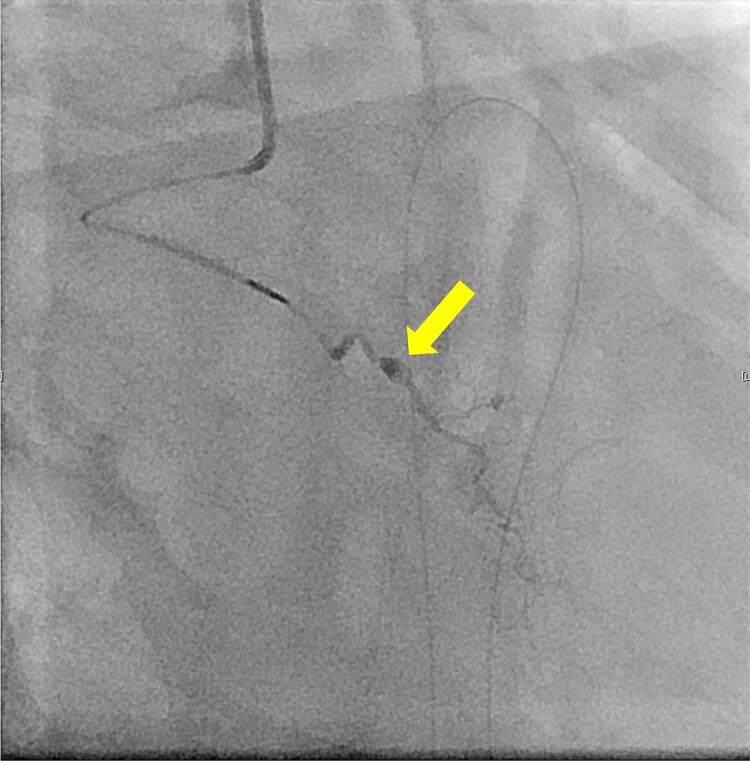
Coronary angiography reveals aneurysmal changes in the right ventricular branch without evident bleeding (arrow).

The patient underwent robotic subtotal esophagectomy, two-region lymph node dissection, narrow gastric tube was created by hand assisted laparoscopy and cervical anastomosis was performed via the retrosternal route. The operation took 613 min, with minimal blood loss. The retrosternal route under camera assist was created without bleeding, and no intraoperative hemodynamic changes or complications were observed. The pathological stage was pT1b(SM2)pN1M0 pStage II (Japanese Classification of Esophageal Cancer, 12th Edition). Esophageal fluoroscopy on postoperative day (POD) 7 showed a minor leak at the anastomotic site, which improved by POD 14 after cervical wound release and drainage. The minor leak at the anastomosis site has been the cause of prolonged hospital stay. The patient experienced sudden intermittent chest pain on POD 17, but his vital signs remained stable. Echocardiography revealed an increased amount of pericardial fluid. He was diagnosed with pericarditis and prescribed colchicine by cardiologist; however, pericardial fluid levels continued to increase. Pericardiocentesis with pericardial catheter insertion was performed on POD 34, revealing bloody pericardial fluid. Blood drainage persisted at approximately 300 ml/h. Urgent contrast-enhanced CT revealed contrast extravasation from the anterior right ventricle (Fig. 2). Coronary angiography revealed aneurysmal changes in the peripheral right ventricular branch without apparent hemorrhage (Fig. 3).

The patient subsequently developed cardiac tamponade and shock. Due to the urgency of the situation, there was no time to consider endovascular treatments such as coil embolization. Consequently, emergency surgery was deemed necessary.

**Figure 4:**
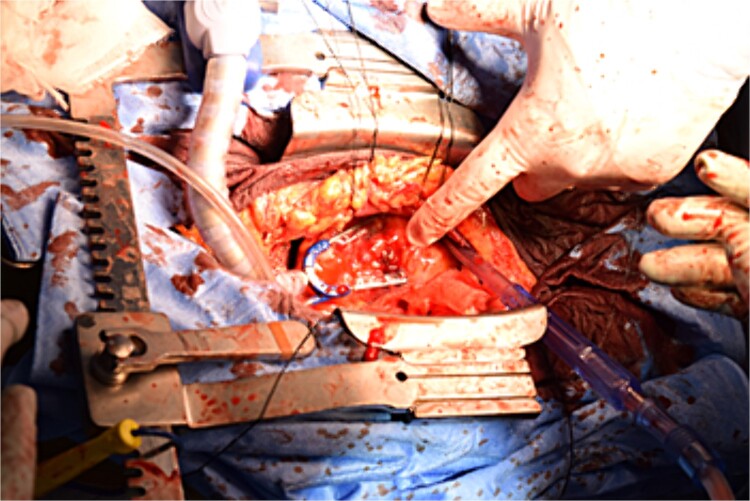
A 5 mm aneurysm in the peripheral right ventricular branch with pulsatile bleeding is observed.

**Figure 5:**
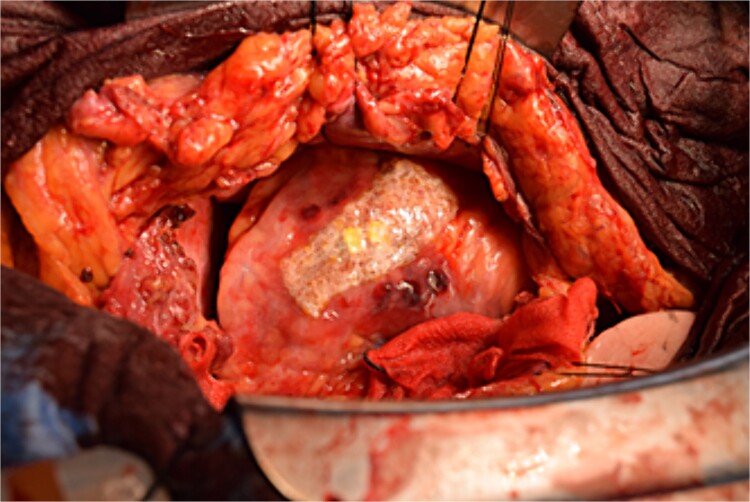
Hemostasis of the coronary artery aneurysm is achieved using horizontal mattress suturing and application of TacoSeal® and Volheal®.

Immediately prior to surgery, he suffered cardiac arrest requiring cardiopulmonary resuscitation but recovered quickly, and a median sternotomy was performed expeditiously. Fortunately, no injury to the retrosternal gastric tube occurred. The gastric tube was pushed into the right thorax to expose the pericardial surface. A pericardial hematoma was observed, and pericardiotomy relieved the tamponade. Removal of the hematoma revealed a 5 mm aneurysm in the peripheral right ventricular branch with pulsatile bleeding (Fig. 4). Hemostasis was achieved using horizontal mattress suturing between the central and peripheral areas of the aneurysm without bypass grafting to the right ventricular branch artery by cardiac surgeon. In addition, TacoSeal® (CSL Behring, Pennsylvania, USA) and Volheal® (KM Biologics, Kumamoto City, Japan) were applied (Fig. 5). The operative time was 133 min, with a blood loss of 1160 ml. Oral intake was initiated on POD 5, and the patient was discharged on POD 22 with an unremarkable postoperative course.

## Discussion

We describe a case of cardiac tamponade caused by rupture of a coronary aneurysm. Cardiac tamponade after esophageal surgery is not commonly encountered [2].

Aoyama *et al*. reviewed cases of cardiac tamponade after esophagectomy [3]. This can occur via two mechanisms: extrapericardial compression, which, although not technically cardiac tamponade, can be caused by dilatation of the reconstructed gastric tube and chylothorax; and intrapericardial fluid collection, which can be caused by acute endocarditis, chylous fistula of the pericardium, and intrapericardial hemorrhage. Cases were managed with percutaneous or surgical drainage, proving that successful management is possible with prompt treatment. The review identified three cases of cardiac tamponade associated with intrapericardial hematoma [3]. In two of the three cases, the cause of the hematoma was not discussed, and in the remaining case, it was noted that an anatomical abnormality caused the anterior surface of the right ventricle to be just below the sternum, contributing to the injuries that occurred during the creation of the retrosternal pathway [3]. These cases had an early onset between POD 0 and 4 [3]. However, in our case, no anatomical abnormalities were observed in the mediastinum, and the cardiac tamponade progressed slowly. Chest pain developed on POD 17, with no hemodynamic changes until pericardiocentesis on POD 34. One possible explanation is that the hematoma provided temporary hemostasis after aneurysm rupture, but bleeding resumed when the pressure in the pericardial sac was lowered by pericardiocentesis. Although not previously reported, coronary aneurysm rupture should be considered as a potential cause of cardiac tamponade after esophageal cancer surgery.

In our case, median sternotomy was performed, allowing easy cessation of bleeding from the anterior right ventricle. In patients with retrosternal reconstruction, median sternotomy carries the risk of gastric tube injury and drainage of cardiac tamponade can easily be achieved via a left anterior thoracotomy [4, 5]. However, visualization of a right-sided bleeding point is challenging with a left thoracotomy, making hemostasis difficult to achieve. A case requiring open heart surgery after esophagectomy via a median sternotomy has also been reported [6]. Despite the risk of gastric tube injury after esophagectomy with retrosternal reconstruction, a median sternotomy should be performed for rapid hemostasis of the right ventricular anterior wall.

A coronary artery aneurysm is defined as a dilation exceeding 1.5 times the width of the normal adjacent coronary artery segment. Coronary artery aneurysms usually cause few symptoms; rupture is rare, but can be lethal. The global prevalence of coronary artery aneurysms is 0.35%. Most patients are male (78.5%), with a mean age of 65 years and cardiovascular risk factors [7]. Regarding revascularization, an interventional approach may be safer and more effective in the long term compared to coronary bypass surgery. In cases with a high risk of coronary aneurysm, we may consider coronary angiography and interventional therapy before surgery to ensure a safer surgical procedure.

In our case, coronary artery aneurysm was thought to be present preoperatively. Although we cannot completely rule out the possibility that damage during intraoperative manipulation may have adversely affected the coronary aneurysm, we do not believe that it was the direct cause, at least because there was no obvious surgical damage to the pericardial side under the speculum. By performing the endoscopic surgery including robot-assisted surgery, it may rather protect against damage to the pericardium by allowing the surgeon to carefully follow the proper layer during the reconstruction procedure.

We encountered a case of a ruptured coronary aneurysm causing cardiac tamponade after esophageal cancer surgery. The median sternotomy approach, although invasive and may potentially damage the gastric tube, is an effective technique for rapid hemostasis.

## Data Availability

Data supporting the findings of this study are available upon request from the corresponding author.
